# Clinical analysis of percutaneous endoscopic unilateral laminotomy for bilateral decompression for single segment degenerative lumbar spinal stenosis: a systematic review and single-arm meta-analysis

**DOI:** 10.3389/fsurg.2025.1458366

**Published:** 2025-02-17

**Authors:** Tianqi Jiang, Zhijun Chen, Yitong Luo, Xinyue Tian, Yanni Zhou, Yanqiang Huan, Yongxiong He

**Affiliations:** ^1^ Graduate School of Inner Mongolia Medical University, Hohhot, Inner Mongolia Autonomous Region, China; ^2^ Department of Orthopedics, Inner Mongolia People’s Hospital, Hohhot, Inner Mongolia Autonomous Region, China; ^3^ School of Chinese Materia Medica, Beijing University of Chinese Medicine, Beijing, China; ^4^ Department of Orthopedics, Beijing Chest Hospital, Capital Medical University, Tongzhou, Beijing, China

**Keywords:** lumbar spinal stenosis, single segment, LSS, percutaneous endoscopic, unilateral laminotomy for bilateral decompression, ULBD

## Abstract

**Background:**

In recent years, percutaneous endoscopic unilateral laminotomy for bilateral decompression (PE-ULBD) has been used to treat degenerative lumbar spinal stenosis (LSS) and has achieved good results. Some researchers have conducted statistical analysies and evaluated the efficacy of this technology. In this systematic review and single-arm meta-analysis, the effectiveness of PE-ULBD as a surgical method for treating single segment LSS was evaluated from the perspective of evidence-based medicine. The aim was to provide a scientific basis for the clinical application of this technology in LSS treatment.

**Methods:**

A systematic review was performed in accordance with the Preferred Reporting Items for Systematic Reviews and Meta-Analyses (PRISMA) guidelines. A total of 396 studies published before May 29, 2024 were collected from the PubMed, Web of Science, Embase, Cochrane Library, China National Knowledge Infrastructure(CNKI), and WanFang databases.

**Results:**

Eight retrospective studies were found with 287 patients who met the inclusion criteria set for the systematic review and single-arm meta-analysis. We used the methodological index for non-randomized studies (MINORS) scale to evaluate the quality of the included studies. The results indicated that significant difference in VAS scores between preoperative and postoperative back and leg pain and the difference between the control results recorded before and after the two types of pain scores was statistically significant (*P* < 0.05). In addition, the difference between the Oswestry Disability Index (ODI) scores recorded in the different groups before and after surgery was statistically significant (*P* < 0.05). Although the results showed high heterogeneity, a sensitivity analysis showed that there was no significant deviation in other results except for the VAS and ODI score for leg pain in the preoperative and three-month postoperative groups. Secondary clinical outcomes included an average operational time of 97.15 min (95% CI = 82.83, 111.47), an average intraoperative bleeding volume of 26.52 ml (95% CI = 10.51, 42.52), an average hospital stay of 4.16 days (95% CI = 2.96, 5.35), and an incidence of complications of 0.10 (95% CI = 0.06, 0.14).

**Conclusion:**

Our results indicate that the PE-ULBD technique has significant short and long-term clinical efficacy for the treatment of single-segment LSS and is worthy of clinical application and promotion.

## 1 Introduction

Lumbar spinal stenosis (LSS) is a prevalent spinal disorder that compresses nerve roots, results in lower back and leg pain, and leads to neurogenic claudication (1). The most common type is degenerative LSS. LSS occurs frequently among the elderly, significantly affecting their physical and mental health. The preferred treatment can be variable based on clinical presentation and other factors. for most LSS patients. However, if severe neurological symptoms persist despite the use of conservative methods, surgery is not “necessary” but it can provide relief and is an option for the patient (2, 3). While performing a traditional posterior lumbar laminectomy can result in the effective removeale of bony structures causing stenosis, a thickened ligamentum flavum, and protruding intervertebral discs (4), as our understanding of degenerative lumbar diseases has progressed, spinal surgeons have discovered that unilateral spinal canal decompression is inadequate for resolving bilateral limb symptoms. In 1988, a England surgeon named Young proposed the unilateral laminotomy for bilateral decompression (ULBD) technique as an alternative to extensive decompression surgery (5). The ULBD technique is designed to minimize the postoperative instability caused by open surgeries. Unlike an extended laminectomy, this surgery preserves the spinous process, contralateral vertebral lamina, and most of the posterior column structure.

When ULBD is performed via traditional open surgery, the paraspinal muscles and posterior ligamentous complex (PLC) are still damaged, causing significant iatrogenic damage and instability. As a result, there is an interest in findings ways to reduce iatrogenic injury while performing precise nerve root and dural sac decompression. In recent years, percutaneous endoscopic approach has been widely used for the treatment of lumbar degenerative diseases such as LSS and lumbar disc herniation, as it causes minimal trauma and achieves thorough decompression (6, 7). Hence, the application of percutaneous endoscopic unilateral laminotomy for bilateral decompression (PE-ULBD) is a major innovation in the treatment of LSS (8), combining percutaneous endoscopic and ULBD technology. By grinding off the upper and lower vertebral lamina and, spinous process roots, and decompressing the areas covered by the ligamentum flavum, this surgical approach achieves decompression of the central spinal canal and bilateral lateral recess. Not only can damage to the stable structure behind the nerve root be avoided, but the recovery of nerve root pulsation has been observed through endoscopy. Furthermore, some researches have found that PE-ULBD has been proven to be effective and safe (9, 10).

The aim of this study was to evaluate the effectiveness of PE-ULBD as a surgical method for treating single-segment LSS using evidence-based medicine. Toward this aim, strict inclusion and exclusion criteria were set for articles to be examined in a systematic review and single-arm meta-analysis. Eight articles were selected that satisfied the criteria. At the same time, this article evaluates the efficacy of PE-ULBD in treating LSS by comparing the differences in eight parameters before and after surgery, including VAS, ODI, surgical time, intraoperative bleeding, hospital stay, and complications. The findings of is study provide a scientific basis for the clinical application of PE-ULBD in the treatment of LSS.

## 2 Materials and methods

### 2.1 Study selection and search strategy

Electronic databases PubMed, Web of Science, Embase, Cochrane Library, CNKI, and WanFang, were searched for all relevant studies until May 29, 2024. Studies were sought that evaluated the use of PE-ULBD as a treatment option for single segment LSS in randomized controlled trials(RCTs), non-RCT, or single-arm clinical studies. A combination of relevant theme words and free words was used to refine the search. After conducting preliminary searches, a search strategy was developed and search terms were adjusted based on the specific database being used. To broaden the search scope and improve accuracy, a selected search strategy combining keywords and subject terms was used to optimize the results. Briefly, the initial search strategy mainly included keywords such as “lumbar spinal stenosis,” “degenerative lumbar spinal stenosis,” “degenerative lumbar central canal stenosis,” “endoscopy,” “ULBD,” “unilateral laminotomy for bilateral decompression,” and “unilateral laminotomy and bilateral decompression.” Two independent reviewers initially screened all the relevant literature identified based on the title and/or abstract; a further round of screening was performed based on the inclusion and exclusion criteria. Differences in opinion between the reviewers were resolved through discussion with the involvement of a third reviewer.

### 2.2 Inclusion criteria

Studies that had the following characteristics were eligible for inclusion in the meta-analysis:

(1) design type: RCT, non-RCT, or single-arm clinical research study on the treatment of LSS with spinal endoscopy; (2) research object: imaging diagnosis of single-segment degenerative LSS accepted due to clear signs of nerve injury in both lower limbs and ineffective conservative treatment; (3) intervention measure: all LSS patients underwent PE-ULBD surgical treatment; (4) outcome measures: the primary outcome measures were Visual Analogue Scale (VAS) scores for back and leg pain and Oswestry Disability Index (ODI) scores at the preoperative, three-month postoperative and 12-month postoperative timepoints, and the secondary outcomes measures included operative time, intraoperative bleeding volume, hospital stay, and complications (to evaluate the clinical efficacy of PE-ULBD). Each study was also required to have a follow-up period of at least 12 months. Only studies published in peer-reviewed journals were considered for inclusion.

### 2.3 Exclusion criteria

Studies were excluded if they met any of the following criteria:

(1) repeat publication or repeat use of case data from the same period and same center; (2)written in a language other than Chinese or English; (3) case report, comment, literature review, thesis, or conference abstract; (4) included individuals with lumbar spondylolisthesis, scoliosis, severe osteoporosis, lumbar tuberculosis, cardiovascular and cerebrovascular diseases, mental illness, or malignant tumors; and (5) included LSS patients treated with the large channel lumbar endoscopic technique.

### 2.4 Data extraction

Two authors independently screened the literature; they reviewed the title, abstract, and full text of each article against the inclusion and exclusion criteria. The corresponding author, Huan, supervised the entire screening process and resolved any discrepancies. In cases where information was incomplete, attempts were made to contact the authors for additional information. The extracted data included:

(1) general information(i.e., lead author, publication year and research type), (2) the participants' characteristics (i.e., sample size, age, level involved, and follow-up time), (3) relevant outcome indicators and measurements, and (4) information on the assessment of the risk of bias in the relevant data.

### 2.5 Quality assessment

In this study, we selected the methodological index for non-randomized studies (11) (MINORS) to evaluate the risk of bias. Because a single set of continuous variable data was evaluated in this study, only the first eight items of MINORS were used for the evaluation.

### 2.6 Outcome indiactor

Due to the limited sample size, the main outcomes of this single-arm meta-analysis were the VAS scores for back and leg pain and the corresponding ODI scores, measured at the same time points. The secondary outcomes of the study included average surgical duration, intraoperative bleeding volume, hospital stay, and complications.

### 2.7 Statistical analysis

The Stata 15.1 (Stata Corp, College Station, TX, USA) software program was used for the statistical analysis. The I^2^ test was utilized to test the heterogeneity of the included literature. According to the Cochrane Handbook, an I^2^ value > 50% indicates heterogeneity, and an I^2^ value < 50% indicates that there is no heterogeneity. A random-effects model was selected for joint statistical analysis, and a fixed-effects model was selected for data processing and analysis. Due to the relatively small incidence of complications reported in the studies included in this meta-analysis (0 < *P* < 0.2), Metaprop was used to perform a double anti-sine transformation of the data, and then statistical consolidation was performed. Additionally, we performed a sensitivity analysis on the outcomes of our meta-analysis. Egger's test was employed to assess the presence of publication bias. A *P*-value < 0.05 was considered statistically significant.

### 2.8 Ethics approval statement

This study did not require the approval of an ethics committee.

## 3 Results

### 3.1 Search results

**Figure 1:**
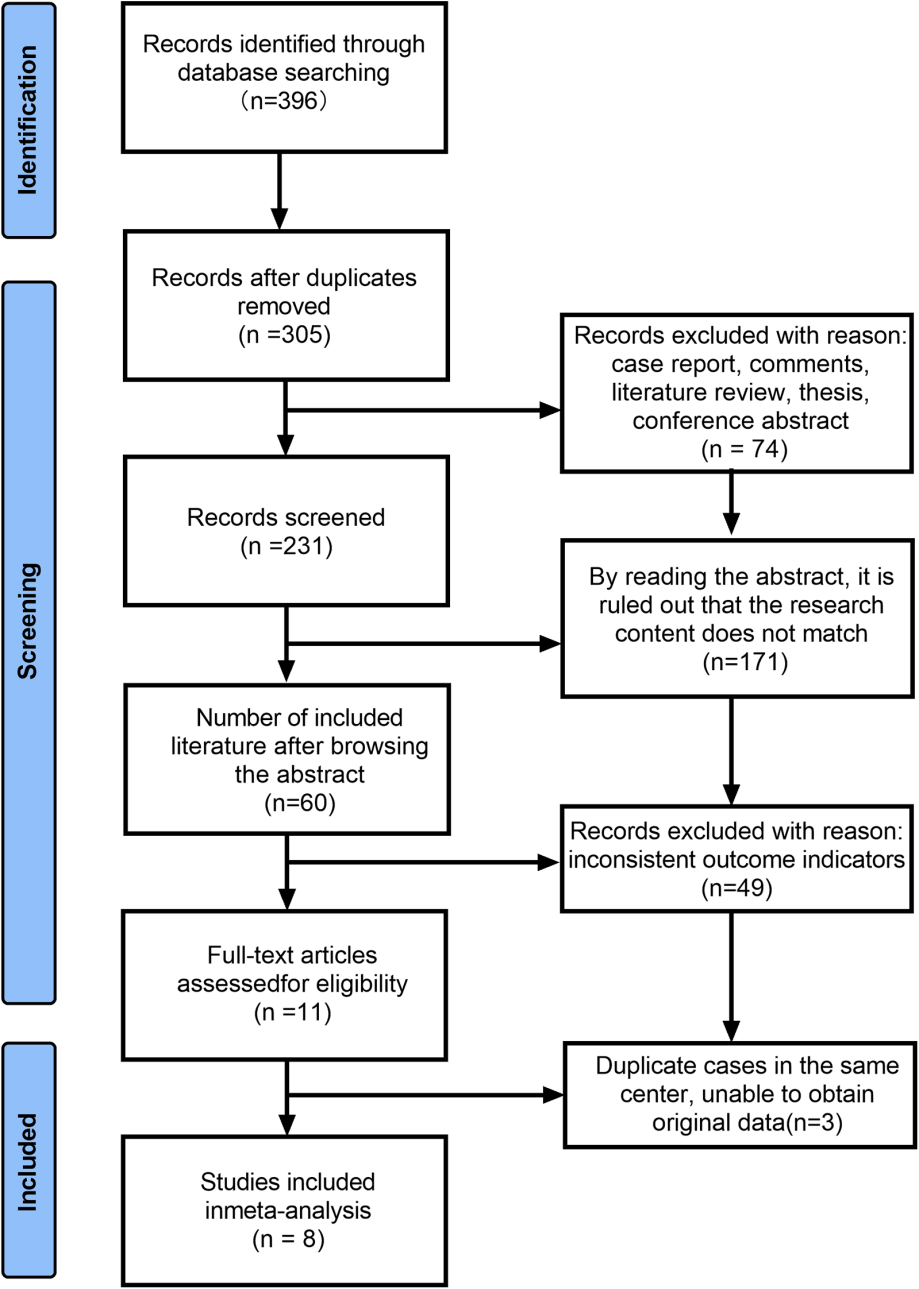
Flow chart of the process used to identify and select studies for this analysis.

The results of the literature search, which was conducted using the aforementioned strategy, are presented in Figure 1. A total of 396 articles were initially identified. Among these, only eight articles that met the inclusion criteria were deemed eligible for further analysis, comprising a total study population of 287 patients.

### 3.2 Study characteristics and quality assessment

The eight studies included in this analysis were retrospective in nature and conducted in China. Table 1 displays the basic characteristics of the studies. Additionally, Table 2 presents the results of the bias risk assessment for the included studies.

**Table 1:** Basic characteristics of included studies.

Reference	Country/region	Design	Sample size(M/F)	Age (years)	Level involved (n)	Follow up time (months)	Operative time (min)	Intraoperative bleeding volume (ml)	Hospital stay (d)
Hua et al. (12)	China	Retrospective	32（12/20）	56.7 ± 9.1	0/3/20/9	≥24	139.5 ± 31.2	51.9 ± 10.9	2.7 ± 0.9
Hua et al. (13)	China	Retrospective	36（14/22）	56.7 ± 8.9	0/3/24/9	>12	137.4 ± 30.1	Not mentioned	2.8 ± 0.9
Meng et al. (14)	China	Retrospective	39（18/21）	59.9 ± 9.4	0/5/22/12	14.1 ± 2.6	82.1 ± 12.3	14.5 ± 5.9	3.7 ± 1.1
Xin et al. (15)	China	Retrospective	47（21/26）	63.4 ± 10.4	0/6/23/18	18.33 ± 4.16	91.17 ± 16.81	13.53 ± 4.94	1d
Bao et al. (16)	China	Retrospective	20（12/8）	Average 68.4	0/0/27/15	>24	Average 75	Not mentioned	Not mentioned
Lv and Mei (17)	China	Retrospective	39（25/14）	66.7 ± 9.2	0/0/9/11	>24	71.2 ± 9.5	Not mentioned	Not mentioned
Xin et al. (18)	China	Retrospective	42（18/24）	Average 61.7	2/11/23/3	Average 18.8	Average 98.2	Not mentioned	Not mentioned
He et al. (19)	China	Retrospective	32（15/17）	62.5 ± 8.37	0/5/17/10	≥12	67.81 ± 5.0	Not mentioned	7.9 ± 2.73

Levels involved: L2-3/L3-4/L4-5/L5-S1.

**Table 2:** Results of risk assessment of bias in included studies.

Reference	A clearly state aim	Inclusion of consecutive patients	Prospective collection of data	Endpoints appropriate to the aim of the study	Unbiased assessment of the study endpoint	Follow-up period appropriate to the aim of the study	Loss to follow up less than 5%	Prospective calculation of the study size	Total score
Hua et al. (12)	2	2	2	2	2	1	2	0	13
Hua et al. (13)	2	2	2	2	2	1	2	0	13
Meng et al. (14)	2	2	2	2	2	2	2	0	14
Xin et al. (15)	2	2	2	2	2	2	2	0	14
Bao et al. (16)	2	2	2	2	2	1	2	0	13
Lv and Mei (17)	2	2	2	2	2	1	0	0	11
Xin et al. (18)	2	2	2	2	2	2	2	0	14
He et al. (19)	2	2	2	2	2	2	2	0	14

The items are scored 0 (not reported), 1 (reported but inadequate) or 2 (reported and adequate).

### 3.3 Meta-analyses

**Figure 2:**
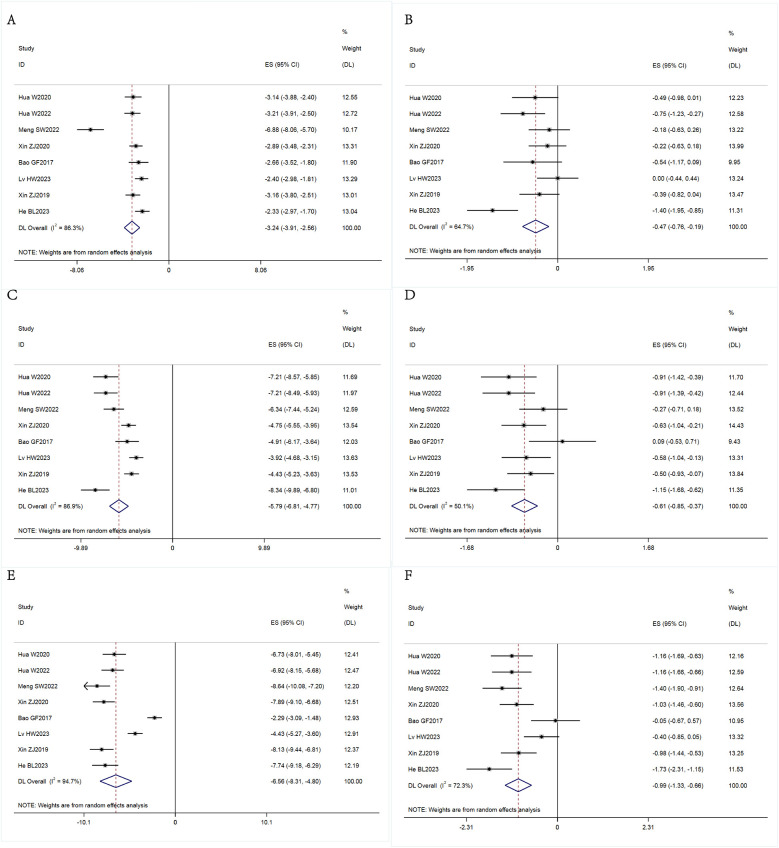
Forest plots of the Visual Analogue Scale (VAS) pain and Oswestry Disability Index (ODI) scores. **(A)** Comparation of VAS scores of patients with back pain at the preoperative and three-month postoperative. **(B)** Comparation of VAS scores of patients with back pain at the three-month postoperative and 12-month postoperative. **(C)** Comparation of VAS scores of patients with leg pain at the preoperative and three-month postoperative. **(D)** Comparation of VAS scores of patients with leg pain at the three-month postoperative and 12-month postoperative. **(E)** Comparation of ODI scores of patients at the preoperative and three-month postoperative. **(F)** Comparation of ODI scores of patients at the three-month postoperative and 12-month postoperative. *P < 0.05 was considered indicative of a statistically significant contribution to the heterogeneity of the effect.

**Figure 3:**
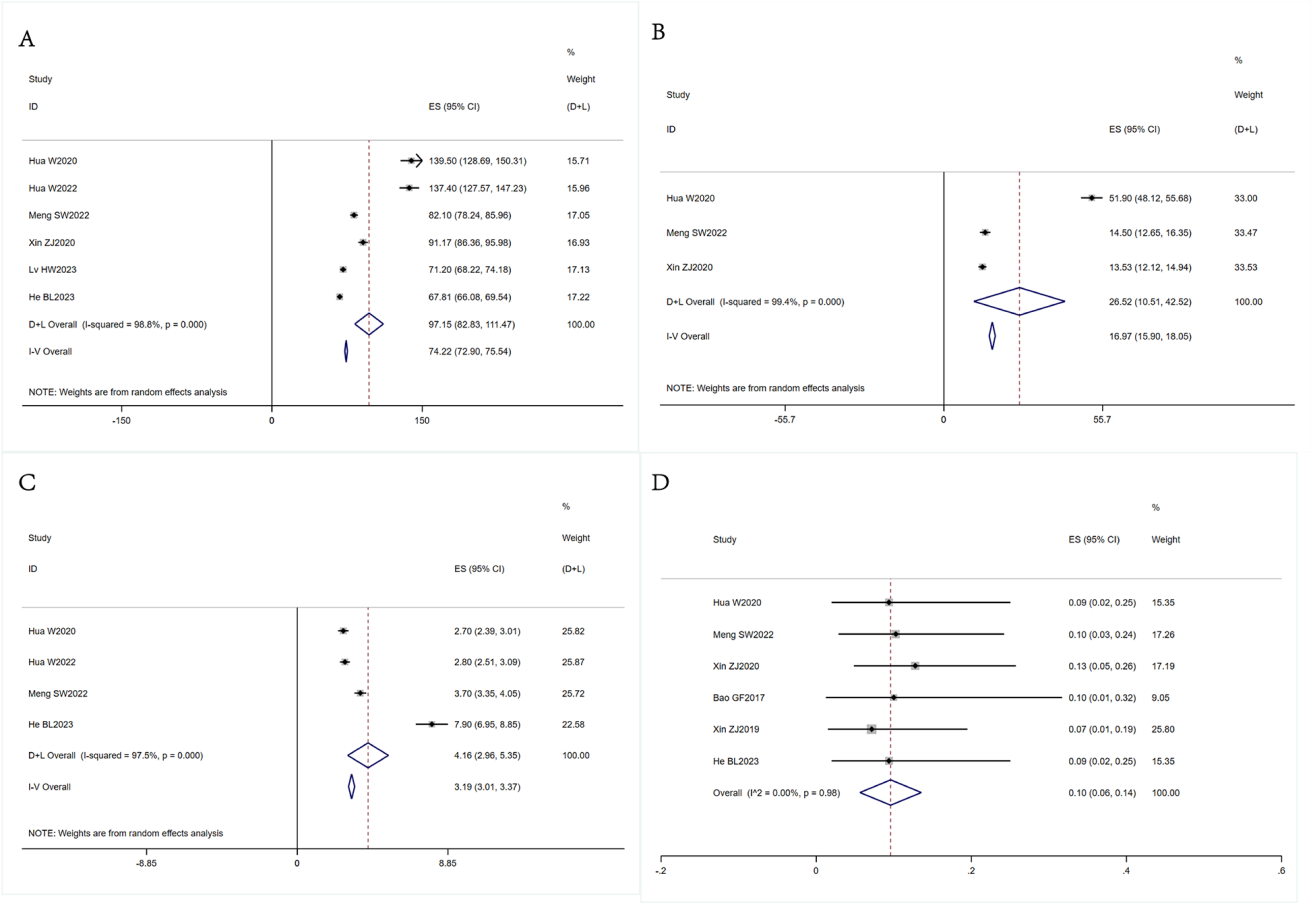
Forest plots of the operative time, intraoperative bleeding volume, hospital stay, and complications. **(A)** The analysis of the operative time. **(B)** The analysis of the intraoperative bleeding volume. **(C)** The analysis of the hospital stay. **(D)** The analysis of the operative time. *P < 0.05 was considered indicative of a statistically significant contribution to the heterogeneity of the effect.

We conducted a meta-analysis to compare the main outcomes, including the pretreatment and posttreatment VAS and ODI scores. The results of our analysis are presented in Figure 2 and Table 3. Additionally, we conducted a meta-analysis to examine the duration of surgery, intraoperative bleeding, length of hospitalization, and incidence of complications. The results of this analysis are depicted in Figure 3 and Table 3.

**Table 3:** Clinical analysis of percutaneous endoscopic unilateral for bilateral decompression for single-segment lumbar spinal stenosis.

Outcomes	Effect	95% CI	P	I^2^(%)	Model
Back pain VAS					
Preoperative/Three-month postoperative	3.24	（3.91, 2.56）	<0.05*	86.3	Random
Three-month postoperative/12-month postoperative	0.47	(0.19, 0.76)	<0.05*	64.7	Random
Leg pain VAS					
Preoperative/Three-month postoperative	5.79	（4.77, 6.81）	<0.05*	86.9	Random
Three-month postoperative/12-month postoperative	0.61	（0.37, 0.85）	<0.05*	50.1	Random
ODI					
Preoperative/Three-month postoperative	6.56	（4.80, 8.31）	<0.05*	94.7	Random
Three-month postoperative/12-month postoperative	0.99	（0.66, 1.33）	<0.05*	72.3	Random
Operative time	97.15	（82.83, 111.47）	<0.05*	98.8	Random
Intraoperative bleeding volume	26.52	(10.51, 42.52)	<0.05*	99.4	Random
Hospital stay	4.16	（2.96, 5.35）	<0.05*	97.5	Random
Complications	0.10	(0.06, 0.14)	>0.05	0	Fixed

* *P* < 0.05 was considered statistically significant contributions to the heterogeneity of effect.

#### 3.3.1 Back pain

We tested for heterogeneity among the eight selected articles and found considerable heterogeneity in the VAS scores of patients with back pain at the preoperative and three-month postoperative time points (I^2^ = 86.3% > 50%). Therefore, we opted for a random-effects model in our meta-analysis. Our results indicated that the VAS score for back pain at three months post-PE-ULBD was lower than the preoperative score, with an effect size of 3.24 [95% confidence interval (CI) = 2.56, 3.91] and a statistically significant outcome (*P* < 0.05). Comparing the VAS scores for back pain measured at the three-month and 12-month postoperative time points revealed that there was high heterogeneity between these values (I^2^ = 64.7% > 50%). Thus, a random-effects model was used for the meta-analysis. Our analysis revealed that the back pain VAS score at the 12-month postoperative time point was lower than the score at the three-month postoperative time point, with an effect size of 0.47 (95% CI = 0.19, 0.76), which was statistically significant (*P* < 0.05) (Figure 2A,B).

#### 3.3.2 Leg pain

Based on heterogeneity testing, we found that the I^2^ value of the VAS scores for leg pain at the preoperative and three-month postopertive time points was 86.9% > 50%. This indicates that there was considerable heterogeneity in the results for this parameter within the selected literature. We subsequently conducted a meta-analysis using a random-effects model, which showed that the VAS score for leg pain at the three-month postoperative time point was slightly lower than the preoperative score, with an effect magnitude of 5.79 (95% CI = 4.77, 6.81). These results were statistically significant (*P* < 0.05). The heterogeneity of the VAS scores for leg pain at the three-month and 12-month postoperative time points was high (I^2^ = 50.1% > 50%). Analysis of the data using a random-effects model revealed that the VAS score for leg pain at 12 months after surgery was lower than the preoperative score, with an effect magnitude of 0.61 (95% CI = 0.37, 0.85). These findings were statistically significant (*P* < 0.05) and are shown in Figure 2C,D.

#### 3.3.3 Oswestry disability index

The preoperative and postoperative ODI scores demonstrated that there was considerable heterogeneity in the data (I^2^ = 94.7% > 50%). A random-effects meta-analysis revealed that there was a statistically significant decrease in the ODI scores three months postoperatively, with an effect size of 6.56 (95% CI = 4.80, 8.31; *P* < 0.05). The selected literature analyzed in this study displayed strong heterogeneity (I^2^ = 62.3% > 50%). Therefore, a random-effects model was selected to conduct the meta-analysis. The consequent random-effects meta-analysis revealed that there was a statistically significant decrease in the ODI scores measured at 12 months postoperatively compared to the scores measured three months postoperatively, with an effect size of 0.99 (95% CI = 0.66, 1.33; *P* < 0.05) (Figure 2E,F).

#### 3.3.4 Operative time

The analysis of the operative time included six articles (12–15, 17). Heterogeneity testing indicated that there was strong heterogeneity (I^2^ = 98.8% > 50%). Thus, a random-effects model was used for the analysis, and it was revealed that the average operative time was 97.15 min (95% CI = 82.83, 111.47) (Figure 3A).

#### 3.3.5 Intraoperative bleeding volume

Our analysis of the intraoperative bleeding volume included three articles (12, 14, 15). Heterogeneity testing revealed that there was considerable variation in the data (I^2^ = 99.4% > 50%), which prompted us to use a random-effects model for the analysis. Our findings indicated that the average intraoperative bleeding volume was 26.52 ml (95% CI = 10.51, 42.52) (Figure 3B).

#### 3.3.6 Hospital stay

Four articles were included in our analysis of the length of hospital stay (12–14). The statistical analysis revealed that there was notable heterogeneity (I^2^ = 97.5% > 50%); therefore, a random-effects model was applied for the analysis. The mean length of hospital stay was 4.16 days (95% CI = 2.96, 5.35) (Figure 3C).

#### 3.3.7 Complications

After excluding three articles that did not provide sufficient details about the occurrence of complications, a total of five articles were included in our analysis of complications (12, 14–16, 18). Our results indicated that there was no heterogeneity (I^2^ = 0 < 50%). Using a fixed-effects model for the analysis, we found that the incidence of complications for PE-ULBD was 0.10 (95% CI = 0.06, 0.14)(Figure 3D).

### 3.4 Sensitivity analysis

**Figure 4:**
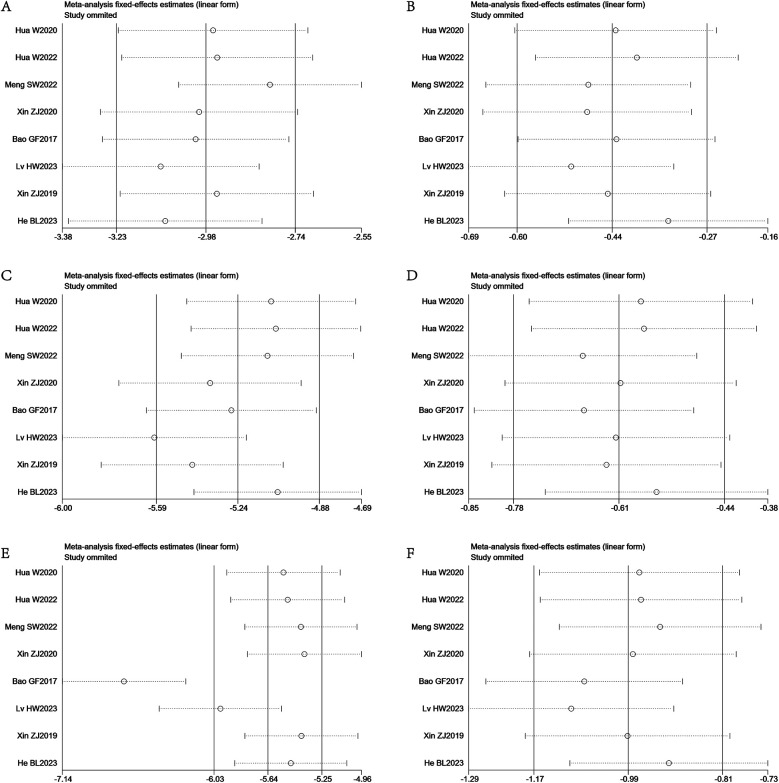
Sensitivity analysis of the VAS pain and ODI scores. **(A)** Sensitivity analysis of VAS scores of patients with back pain at the preoperative and three-month postoperative. **(B)** Sensitivity analysis of VAS scores of patients with back pain at the three-month postoperative and 12-month postoperative. **(C)** Sensitivity analysis of VAS scores of patients with leg pain at the preoperative and three-month postoperative. **(D)** Sensitivity analysis of VAS scores of patients with leg pain at the three-month postoperative and 12-month postoperative. **(E)** Sensitivity analysis of ODI scores of patients at the preoperative and three-month postoperative. **(F)** Sensitivity analysis of ODI scores of patients at the three-month postoperative and 12-month postoperative.

We conducted a sensitivity analysis, and the results showed that only the Lv HW2023 study on VAS score grouping of patients according to leg pain before and three months after surgery and the Bao GF2017 study on ODI score grouping of patients before and three months after surgery had a significant impact on the results. The sensitivity analysis results are shown in Figure 4.

### 3.5 Bias test

**Figure 5:**
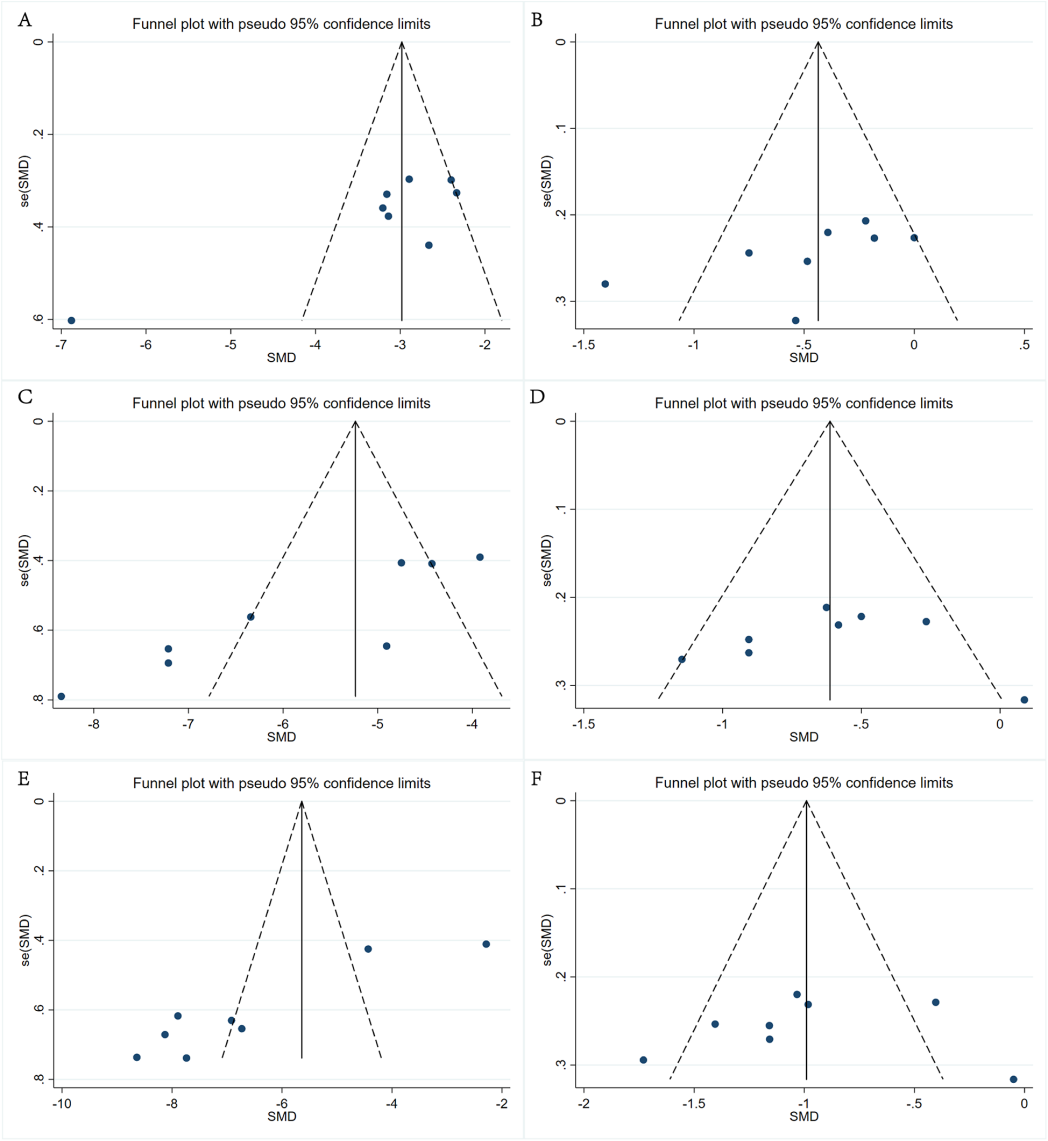
Funnel plot of the VAS pain and ODI scores. **(A)** Back pain VAS scores recorded preoperatively and three months postoperatively, **(B)** back pain VAS scores recorded three months and 12 months postoperatively, **(C)** leg pain VAS scores recorded preoperatively and three months postoperatively, **(D)** leg pain VAS scores recorded three months and 12 months postoperatively, **(E)** ODI scores recorded preoperatively and three months postoperatively, and **(F)** ODI scores recorded three months and 12 months postoperatively.

We generated a funnel plot (Figure 5) to assess the publication bias in the VAS and ODI scores and used Egger's test to determine the degree of asymmetry in the plot. No significant deviation was observed in the results, except for the VAS and ODI scores for back and leg pain in the preoperative and three-month postoperative groups (*P* > 0.05).

## 4 Discussion

LSS can be classified into three categories based on the location of the stenosis: central canal stenosis, lateral recess stenosis, and intervertebral foramen stenosis. Lateral recess stenosis is the most widespread form of LSS. Currently, surgical methods such as laminectomy and fenestration decompression are extensively used in clinical practice to treat central canal and bilateral lateral recess stenosis. However, at the same time, the drawbacks associated with open surgery cannot be ignored. These drawbacks include high bleeding volume, large trauma, slow recovery, and long hospital stay (20). Numerous studies have demonstrated that the extensive dissection of structures—for instance, the erector spinalis muscle and posterior ligament complex—may result in iatrogenic lumbar instability and chronic postoperative lower back pain (21–24). In addition, with the deepening understanding of degenerative lumbar spine diseases, it has been found that unilateral spinal canal decompression cannot effectively eliminate bilateral limb symptoms. Therefore, to solve the two major problems of large trauma caused by open surgery and incomplete bilateral decompression caused by unilateral decompression, different ULBD surgical methods have been proposed, such as PE-ULBD, microscopic-ULBD, minimally invasive surgery ULBD (MIS-ULBD), unilateral biportal endoscopic ULBD (UBE-ULBD), and microscopic discometry ULBD (MED-ULBD).

### 4.1 Technologies for other types of ULBD

Chen et al. (25) found that in patients treated for single-segment LSS, the estimated blood loss, analgesic use, hospital stay, and VAS score for back pain values in those who underwent PE-ULBD were all less than the values recorded in those who underwent microscopic-ULBD. Meanwhile, Yang et al. (26) observed that in addition to the above advantages, PE-ULBD was more helpful in improving patient pain in the short term. Hasan et al. (27) made the clinical observation that the total number of perioperative medical events in patients who underwent MIS-ULBD was greater than that in patients who underwent PE-ULBD (5 [26%] vs. 2 [8%]), indicating that PE-ULBD was associated with fewer overall adverse events and lower reoperation rates. In addition, compared to tubular distractors, the working sleeve used in percutaneous endoscopy causes less tissue damage due to its smaller diameter and more flexible axial adjustment. Regarding UBE-ULBD, Hua et al. (13) found that the clinical efficacy of PE-ULBD and UBE-ULBD was similar and recommended UBE-ULBD. They concluded that UBE-ULBD can provide a better field of vision and a larger operating space for surgery due to the presence of dual channels. However, due to the repeated entry and exit of surgical instruments through the operating channel, UBE-ULBD may cause more damage to adjacent structures than PE-ULBD. In contrast to the abovementioned surgical methods, due to the intraoperative placement of the endoscope outside the vertebral lamina, MED-ULBD provides a smaller field of view within the spinal canal and a more limited operating range. As a consequence, it is easy to cause incomplete decompression within the spinal canal, which limits the clinical application of this technique to some extent.

### 4.2 Advantages of Pe-ULBD

The advent of PE-ULBD technology offers not only an effective way to address the disadvantages associated with open surgery but also a superior solution that alleviates the severe bilateral limb symptoms caused by bilateral lateral recess stenosis. Compared to traditional surgery and endoscopic-assisted decompression, PE-ULBD has several advantages. First, intraoperative bleeding and the accumulation of inflammatory substances in the spinal canal are minimized by continuous irrigation of the canal with saline. This novel technique guarantees a clear surgical field and effective and complete spinal canal decompression while diminishing the occurrence of dural tears. Second, PE-ULBD can decompress the contralateral narrow area entirely through an inclined working channel, eliminating the need for an inclined operating table to accomplish contralateral decompression. Third, PE-ULBD simplifies the surgical steps and reduces surgical duration. Despite these advantages, the novelty and long learning curve of PE-ULBD have resulted in limited application of this technique. However, Lee et al. (28) stated that the length of the learning curve can help doctors to better understand and master the technology and thus shorten the time required for lumbar surgery and reduce the incidence of complications, which is beneficial to patients.

### 4.3 Results of meta-analysis

The aim of the present study was to summarize the clinical efficacy of PE-ULBD for the treatment of LSS through a meta-analysis to assist the clinical promotion of this technique. The efficacy of PE-ULBD for treating LSS was evaluated using eight parameters, which included the VAS, ODI, operative time, intraoperative bleeding volume, hospital stay, and complications. Eight original studies that encompassed 287 patients were analyzed. These eight studies and their data provide valuable information for subsequent learning and application and are worth developing. In the present study, we assessed the efficacy of PE-ULBD by analyzing the VAS and ODI values recorded before and after surgery. We found that a significant difference in VAS scores between preoperative and postoperative back and leg pain was after surgery and the difference between the control results recorded before and after the two types of pain scores was statistically significant (*P* < 0.05). The results indicated that undergoing PE-ULBD had a significant therapeutic effect; namely, it could effectively relieve stenosis and pain (9, 25, 26). What' more, the comparison of ODI scores between different groups before and after surgery was statistically significant (*P* < 0.05). At the same time, our research suggests significant variability (high I^2^ values) across studies for key outcomes like VAS and ODI scores, operative time, and hospital stay is acknowledged. Heterogeneity often arises from clinical, methodological, and statistical variations. When heterogeneity is present, we can employ subgroup analysis, sensitivity analysis, and meta-regression to investigate its sources. Following our sensitivity analysis, the results indicated that there were no significant deviations in other outcomes, except for the VAS score of leg pain and the ODI score at the preoperative and three-month postoperative time points. The eight studies included in the meta-analysis were all retrospective, indicating that there was no methodological heterogeneity among the included studies. However, significant differences existed due to factors such as variations in the populations studied and the methods used by staff to collect clinical data. These factors contributed to substantial clinical and statistical heterogeneity in the final meta-analysis results. Additionally, the sources of heterogeneity also may be attributed to differences in surgical procedures among the operators. Due to the novelty of this technology, there are significant differences in the skills and experience of doctors, resulting in varying degrees of differences in results. We found through Egger's test on the data that no significant deviation was observed in the results, except for the VAS scores for back and leg pain and ODI in the preoperative and three-month postoperative groups (*P* > 0.05). Overall, PE-ULBD was found to have clinical significance in terms of improving back and leg pain. Although the number of enrolled cases was not large, the results showed that back and leg pain could be significantly alleviated in the short to medium term. Hence, PE-ULBD may be an effective treatment for LSS, and its efficacy may be related to the restoration of the posture balance lost due to back and leg pain and the preservation of more paravertebral muscles and zygapophysial joints.

Regarding the other listed perioperative indicators, in this study we only conducted a single-arm summary analysis, and the results can be used as a reference by clinical physicians. The following results were obtained: the average operational time was 97.15 min (95% CI = 82.83, 111.47), the average intraoperative bleeding volume was 26.52 ml (95% CI = 10.51, 42.52), the average hospital stay was 4.16 days (95% CI = 2.96, 5.35), and the incidence of complications was 0.10 (95% CI = 0.06, 0.14). Overall, our results indicate that PE-ULBD has a significant therapeutic effect for single-segment LSS and is worthy of clinical application and promotion.

### 4.4 Complications

Complications are a crucial factor in evaluating the safety of a surgical procedure and determining its clinical applicability and efficacy. These complications include dural tear, cauda equina nerve injury, nerve root paralysis, postoperative sensory abnormalities, postoperative vertebral instability, transient muscle strength decline, etc. Therefore, it is crucial to develop a reasonable surgical strategy before the operation to prevent these complications. The surgical approach should be determined based on the side with more severe symptoms or imaging-indicated stenosis. As a general principle, the ipsilateral spinal canal should be addressed first, followed by the contralateral spinal canal. During PE-ULBD surgery, it is essential to preserve the ligamentum flavum as much as possible before completing the bony structure treatment to reduce the risk of spinal cord and nerve-related injuries (18). To minimize postoperative vertebral instability, the medial facet joint removal should be kept to less than 50% to reduce the impact on postoperative segmental stability (18). Typically, the learning curve for spinal endoscopy is steep, and most complications occur early in the process (17). It is important to maintain a clear surgical field by continuously flushing with normal saline and rotating and tilting the endoscopic tube during the operation (15, 16). Regarding transient muscle strength decline, Bao et al. suggest that it may be caused by prolonged compression of nerve roots and the dural sac by the working channel (16). Of note, when surgery is performed on segments above L4–5, nerve root irritation is more likely to occur based on our experience; hence, extra care and attention are required in such situations to minimize this irritation. Thus, evaluating the feasibility of surgical methods requires that both the postoperative efficacy and the possible complications that may arise from the surgery are considered. Our research indicates that the incidence of complications resulting from PE-ULBD is relatively low, at 10%. Nonetheless, given the limited number of studies included in this analysis and their small sample sizes, the resultant data cannot be considered fully representative. Thus, further research should be conducted to investigate the types and incidence of complications associated with PE-ULBD and thus enhance the accessibility and efficacy of this surgical method. As more surgeons become more proficient in and master this surgical method, the incidence of complications is likely to decrease.

In terms of the surgical process, the complete removal of the spinous process base is a key step in PE-ULBD, as it facilitates sufficient decompression of the ipsilateral and contralateral crypts. However, this leads to corresponding increases in surgery time and intraoperative bleeding compared to simple spinal endoscopic decompression. For multi-segment LSS, the surgical time will be longer and the amount of bleeding will be greater than for single-segment LSS. Therefore, we do not recommend PE-ULBD for the treatment of patients who are older, have more comorbidities, and cannot tolerate prolonged surgery.

Currently, PE-ULBD is gradually being promoted as a minimally invasive surgical method in clinical practice. However, due to the novelty of this technique and the steep learning curve, there may be some difficulties that hinder its widespread application, which is not very friendly to novice surgeons. Meanwhile, for surgical operators, there are technical issues such as the possibility of intraoperative complications, limited operating space, and incomplete decompression. These are the current challenges associated with this technique and should be the focus and emphasis of future development and promotion of this surgical approach.

### 4.5 Limitations

This study has several limitations. First, there is a need to improve the research quality of the meta-analysis. Only eight non-RCTs were included in this meta-analysis, highlighting the need for further high-quality studies to be conducted to provide more robust evidence. Secondly, due to the small sample size, low data quality, and significant variations in surgical practices, proficiency, operation time, and population characteristics across many studies, there is a high degree of heterogeneity in the primary outcomes included in this article. Therefore, the statistical results of the primary outcomes in this study must be interpreted with caution.Additionally, all of the studies that met the inclusion criteria were conducted by Chinese scholars, and this may limit the generalizability of the results to a broader population. Furthermore, it is important to note that the skills and experience of the surgeon have a significant impact on the incidence of complications. Therefore, caution should be exercised when generalizing these findings. Moreover, there is a need for more RCTs to be conducted with large sample sizes and across multiple centers to validate the current research results.

## 5 Conclusion

The findings of this study indicate that PE-ULBD is clinically effective and safe in the short and long term for treating LSS. However, However, during the treatment of LSS with PE-ULBD, complications such as dural tear, epidural hematoma, spinal instability, and postoperative headache still exist. How to better leverage the advantages of PE-ULBD while avoiding complications is a major challenge for the future. Therefore, it is necessary for spinal surgeonto fully understand the surgical indications before deciding to perform this surgery. On the basis of fully mastering anatomical knowledge, the surgeon is required to have rich experience in spinal endoscopic surgery. Only in this way can we best judge, manage, and minimize surgery-related complications and improve the success rate of surgery. Although the decision to perform a specific surgical procedure to treat LSS is ultimately dependent on the characteristics of the condition and the surgeon's preferences, various studies have shown that PE-ULBD is an effective option with a good risk profile for properly selected patients. Thus, given the current widespread application of endoscopy in spinal surgery, more and higher quality RCTs must be conducted to investigate the applicability of this technology for treating LSS.
